# Tartary buckwheat *FtF3′H1* as a metabolic branch switch to increase anthocyanin content in transgenic plant

**DOI:** 10.3389/fpls.2022.959698

**Published:** 2022-08-25

**Authors:** Chenglei Li, Jingjing Yang, Kai Yang, Huala Wu, Hui Chen, Qi Wu, Haixia Zhao

**Affiliations:** College of Life Science, Sichuan Agricultural University, Ya'an, Sichuan, China

**Keywords:** Tartary buckwheat *(Fagopyrum tataricum)*, metabolism, flavonoid biosynthesis, flavonoid 3′-hydroxylase, transgenic plants

## Abstract

Tartary buckwheat (TB) is a pseudocereal rich in flavonoids, mainly including flavonols and anthocyanins. The flavonoid 3′-hydroxylase (F3′H) is a key enzyme in flavonoid biosynthesis and is encoded by two copies in TB genome. However, its biological function and effects on flavonol and anthocyanin synthesis in TB have not been well validated yet. In this study, we cloned the full-length *FtF3′H1* gene highly expressed in all tissues (compared with *FtF3′H2*) according to TB flowering transcriptome data. The corresponding FtF3′H1 protein contains 534 amino acids with the molecular properties of the typical plant F3′H and belongs to the CYP75B family. During the flowering stage, the *FtF3′H1* expression was highest in flowers, and its expression pattern showed a significant and positive correlation with the total flavonoids (*R*^2^ > 0.95). The overexpression of *FtF3′H1* in *Arabidopsis thaliana*, *Nicotiana tabacum* and TB hairy roots resulted in a significant increase in anthocyanin contents (*p* < 0.05) but a decrease in rutin (*p* < 0.05). The average anthocyanin contents were 2.94 mg/g (fresh weight, FW) in *A. thaliana* (about 135% increase), 1.18 mg/g (FW) in *tobacco* (about 17% increase), and 1.56 mg/g (FW) TB hairy roots (about 44% increase), and the rutin contents were dropped to about 53.85, 14.99, 46.31%, respectively. However, the expression of genes involved in anthocyanin (DFRs and ANSs) and flavonol (FLSs) synthesis pathways were significantly upregulated (*p* < 0.05). In particular, the expression level of *DFR*, a key enzyme that enters the anthocyanin branch, was upregulated thousand-fold in *A. thaliana* and in *N. tabacum*. These results might be attributed to *FtF3′H1* protein with a higher substrate preference for anthocyanin synthesis substrates. Altogether, we identified the basic biochemical activity of FtF3′H1 *in vivo* and investigated its involvement in anthocyanin and flavonol metabolism in plant.

## Introduction

Tartary buckwheat (*Fagopyrum tataricum* Gaertn.) belongs to the *Fagopyrum* genus (Polygonaceae), mainly being cultivated in Asian countries. It is a brilliant cereal with edible and medicinal characteristics. Enriched flavonoids provide the pharmacological activity for TB (Li et al., 2012b; Dong et al., 2020; Park et al., 2021). As a focus in the secondary metabolism of plants, flavonoids not only endow various colors including red, blue, and purple as pigmentation resources in plants, but also are associated with health as active compounds in vegetables and fruits (Grotewold, 2006). In addition, flavonoids are well-known antioxidants that have numerous pharmacologic properties such as anti-inflammatory, anticarcinogenic, antimetastatic, and other effects (Li et al., 2012a).

**Figure 1:**
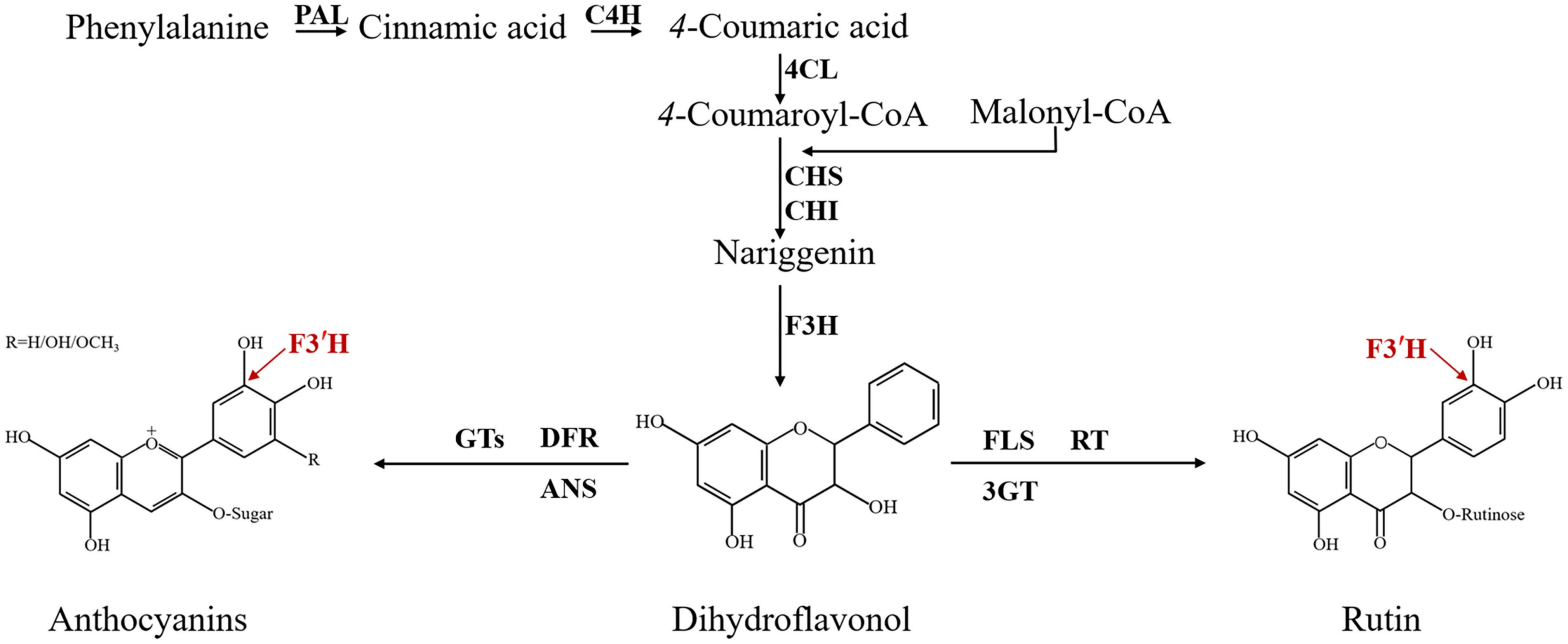
Flavonoid biosynthesis pathway of TB. PAL: phenylalanine ammonialyase; C4H: cinnamate 4-hydroxylase; 4CL: 4-coumarate CoA ligase; CHS: chalcone synthase; CHI: chalcone isomerase; F3H: flavanone 3-hydroxylase; F3′H: flavonoid 3′-hydroxylase; DFR: dihydroflavonol 4-reductase; FLS: flavonol synthase; ANS: anthocyanidin synthase; GTs: glucosyl transferases; 3GT: flavonoid 3-O-glucosyltransferase; RT: 3-O-rhamnosyltransferase.

Since the mid-1980s, biochemical information about flavonoid enzymes has proliferated, and the biosynthesis, accumulation, core enzymes, and genes involved in flavonoid biosynthesis are now widely described in different species through the flavonoid biosynthesis pathway, such as *Arabidopsis thaliana*, *Malus*, *Fragaria vesca*, and so on (Prescott and John, 1996; Han et al., 2010; Thill et al., 2013; Wu et al., 2020). In the model plant *A thaliana*, at least 54 flavonoid molecules are found and more than 30 genes are involved in their biosynthesis (Routaboul et al., 2006). The enzyme-coding genes Phenylalanine ammonia-lyase (*PAL*; Shufflebottom et al., 1993), Cinnamic acid 4-hydroxylase (*C4H*; Mizutani et al., 1997), 4-coumarate CoA ligase (*4CL*; Hamberger and Hahlbrock, 2004), Chalcone synthase (*CHS*; Austin and Noel, 2003), Chalcone isomerase (*CHI*; Winkel-Shirley, 2001), Flavanone 3-hydroxylase (*F3H*; Pelletier and Shirley, 1996), Flavonoid 3′-hydroxylase (*F3′H*; Schoenbohm et al., 2000), Flavonol synthase (*FLS*; Prescott et al., 2002), Dihydroflavonol 4-reductase (*DFR*; Shirley et al., 1992), Leucoanthocyanidin dioxygenase/anthocyanidin synthase (*LDOX/ANS*; Saito et al., 1999; Abrahams et al., 2003), Anthocyanidin reductase (*ANR*; Devic et al., 1999), and Polyphenol oxidase (*PPO*; Pourcel et al., 2007) constitute the central flavonoid biosynthetic pathway in *A thaliana*. The flavonoid 3′-hydroxylase (F3′H) is a vital enzyme in the flavonoid biosynthesis pathway (Figure 1). It belongs to a member of cytochrome P450 monooxygenase (CYP) superfamily, which catalyzes NADPH-dependent hydroxylation of substrates and associates to various metabolic pathways, including flavonoid biosynthesis (Awasthi et al., 2016). The main function of F3′H is to increase water solubility by catalyzing the hydroxylation of C atoms in the B-ring (Grotewold, 2006). CYP encoded by F3′H has been well explored in a variety of plants and its function has been demonstrated. For example, F3′H overproduction induced the F3′H-and F3′5′H-deficient pale-pink *petunia* to produce fuchsia flowers due to the accumulated anthocyanidins (Tanaka, 2006). In *Actinidia* (Peng et al., 2019), *Arachis hypogaea* (Xue et al., 2021), *Brassica rapa* (Park et al., 2021), and other plants, the expression of *F3′H* was strongly correlated with the content of different flavonoids, respectively. Obviously, F3′H is involved in multiple actions and has a significant effect on flavonoid metabolism in plants.

Tartary buckwheat contains distinct types of flavonoids, the most abundant active biological compounds (Peng et al., 2015; Huda et al., 2021; Li et al., 2022). Flavonols, represented by rutin, are the main type of TB flavonoids, accounting for 1.7% of the dry weight of TB grains. Besides, anthocyanins largely accumulate in the TB stems and leaves, thereby strengthening its external stress-resistance (Fabjan et al., 2003; Park et al., 2011). Hence, it is an indispensable process to promote TB flavonoid accumulation and identify the associated key enzyme genes in the biosynthetic flavonoid pathway. To date, studies on TB genome, transcriptome, and metabolome, have provide a new perspective on molecular regulation of flavonoid synthesis in TB (Gao et al., 2016; Yao et al., 2016; Huang et al., 2019; Liu et al., 2019; Hou et al., 2021). However, the biological function of some genes such as *FtF3′H1* has not been identified, which impedes to clarify the metabolic branches of flavonoids synthesis in TB. In our previous work, *FtF3′H1* gene containing the conserved motif (GGER/K) was successfully cloned by RACE. However, the corresponding sequence lacked about 40 amino acids at the N-terminal, thereby resulting in unidentified biological activity (Li et al., 2014). In this study, the complete *FtF3′H* coding sequence was isolated from TB, and its activity and function were analyzed by performing homologous and heterologous expression experiments, respectively. These results will improve the acknowledge of the flavonoid metabolic pathways in TB, subsequently promote the accumulation of natural medicinal metabolites and contribute to the molecular breeding of TB.

## Materials and methods

### Plant materials and growth conditions

The TB cultivar., Xiqiao 2, was provided by Professor An’hu Wang from Xichang University. TB seeds were grown in farm field of Sichuan Agriculture University at Ya’an, Sichuan. TB roots, stems, leaves, flowers, and seeds during plant flowering were collected to investigate the total flavonoid content and relative expression level of *FtF3′H1*. The corresponding samples were frozen in liquid nitrogen, and stored at –80°C.

*Nicotiana tabacum* “NC89” and *Arabidopsis thaliana* ecotype Columbia-0 (Col-0), *tt7* mutant an *Arabidopsis* line lacking *F3′H* gene function were provided by Professor Jinwen Zhang from Gansu Agricultural University and Professor Yi Cai from Sichuan Agricultural University, respectively. *N. tabacum* and *A thaliana* were germinated in a greenhouse with 16 h photoperiod. Additionally, TB seeds were sterilized with 70% ethanol for 90 s and HgCl solution for 10 min and then rinsed several times in sterile water. These seeds were grown on agar plates containing sucrose-free half Murashige and Skoog (½ MS) medium with pH5.8 and 0.8% agar at 25°C with 16 h photoperiod for subsequent hair roots preparation.

### Cloning and sequence analysis of *FtF3'H*

Based on TB genome sequence and transcriptome data (Yao et al., 2017a; Zhang et al., 2018), we obtained the full-length cDNA sequence of *FtF3′H1* by RT-PCR. The amplified PCR fragments were cloned into pMD*^@^*19-T vector and then sequenced. The amino acid sequence of FtF3′H1 was deduced by DNAman software (version 9.0), following with the homology analysis performed using MEGA software (version 7.0). Continually, the genetic structure was predicted by the online software GSD2.0.^1^ The physicochemical characteristics of the deduced FtF3′H1 protein, including molecular formula, relative molecular weight, and isoelectric point were characterized using the online website ExPASy ProtParam tool.^2^ Predicted molecular docking models of FtF3′H1 with substrates were obtained with the MOE (version 2019) software.

### Prokaryotic expression of *FtF3'H1* in *Escherichia coli*

The ORF of *FtF3′H1* was amplified and inserted in to pGEX-4T. Then the recombinant plasmid of pGEX-4 T-*FtF3′H1* was transformed into *E. coli* BL21(DE3). A single colony was inoculated and cultivated in LB medium supplemented with kanamycin (50 μg/ml) at 37°C in the incubator with 220 rpm. When the OD_600_ of the bacterial culture reached to 0.6, 0.2 mmol/l isopropyl *β*-*D*-1-thiogalactopyranoside (IPTG) was added to induce the expression of FtF3′H1 fused with a GST-tag at 18°C for 8 h.

To investigate the potential function of the FtF3′H1 recombinant protein, we performed affinity chromatography to purify it with a BeyoGold^™^ GST labeling Resin kit. Continually, the purified FtF3′H1 was supplemented with 100 mmol/l KH_2_PO_4_-NaOH buffer (pH = 0.8), 20 mmol/l *β*-mercaptoethanol, 1.25 mmol/l NADPH, and then mixed with 100 mmol/l kaempferol and 100 mmol/l dihydrokaempferol as the substrate. Enzyme activity was determined under conditions at 30°C for 1 h, and an equal volume of ethyl acetate was used to extract reaction compounds. Subsequently, the extracted precipitation was dissolved in 100 ml of 80% methanol solution and then determined by High-performance liquid chromatography (HPLC).

### Transgenic plants overexpressing *FtF3'H1*

The *FtF3′H1* ORF was cloned into the constitutive expression vector pCHF3-eYFP driven by 35S promoter. The recombinant plasmid pCHF3*-FtF3′H1-*eYFP was transformed into the strain *Agrobacterium tumefaciens* strains GV3101 and *A4*. GV3101 was used to transform *Arabidopsis* and *N. tabacum*, and *A4* for TB to generate transgenic plants. The transgenic *Arabidopsis* T3 lines were selected on MS medium with 50 mg/ml kanamycin (Huang et al., 2019), and the *N. tabacum* T3 lines were obtained on MS medium with 100 mg/ml kanamycin. Moreover, the *FtF3′H-overexpressing* TB hairy root lines were selected in ½ MS liquid medium with 50 mg/ml kanamycin (Huang et al., 2016).

### Determination of flavonoid and anthocyanin contents in transgenic plants

The flavonoid and anthocyanin were extracted from TB tissues (root, stem, leaf, flower, and seed), transgenic *Arabidopsis* lines, *N. tabacum* flowers and TB hairy roots, respectively, using the method as described previously (Liu and Zhu, 2007; Yao et al., 2017b). The total flavonoid and anthocyanin contents were determined by a spectrophotometry as previously reported (Liu and Zhu, 2007; Zhang et al., 2009). HPLC was performed to determine rutin content according to the reported method (Yao et al., 2020). Three biological repeats were set for each experimental group.

### Quantitative real-time PCR analysis

To determine the expression levels of flavonoid-associated genes in different samples, total RNA was extracted using RNAout Kit (TIANGEN, China). Quantitative real-time PCR (qRT-PCR) was performed using the PreMix Ex *Taq* II kit (Tli RNAseH Plus) and with CFX96 RT-PCR machine (Bio-Rad, United States). The PCR amplification procedure was as follows: 95°C for 10 s, 40 cycles at 95°C for 5 s, 61°C for 30 s. The housekeeping gene *FtH3* (GenBank ID: HM628903 for TB), *β-Actin2* (GenBank ID: AF149413 for *Arabidopsis*), and *β-actin* (GenBank ID: AB158612 for *N. tabacum*) were used as the internal standards, respectively. The analysis of each sample was repeated three times. Relative expression level was calculated using the 2^−ΔΔCt^ method.

### Primers and statistical analysis

All the primer sequences used in this study are shown in Supplementary Table S1. Data were analyzed using the SPSS 19.0 statistical software. Significant differences were indicated when *p* < 0.05.

## Results

### Cloning and sequence analysis of the *FtF3'H1* gene

Based on TB transcriptome data, two different transcripts were annotated as *F3′H*, named *FtF3′H1* and *FtF3′H2*. To investigate the role of two *FtF3′Hs* in flavonoid biosynthesis, we performed a correlation analysis between the expression level and flavonoid content in different tissues of flowering TB. Results illustrated that *FtF3′H2* revealed traces of expression in all TB tissues except roots, however, *FtF3′H1* showed a high expression pattern (Supplementary Figure S1). Thus, *FtF3′H1* was selected as the candidate gene for further characterization.

**Figure 2:**
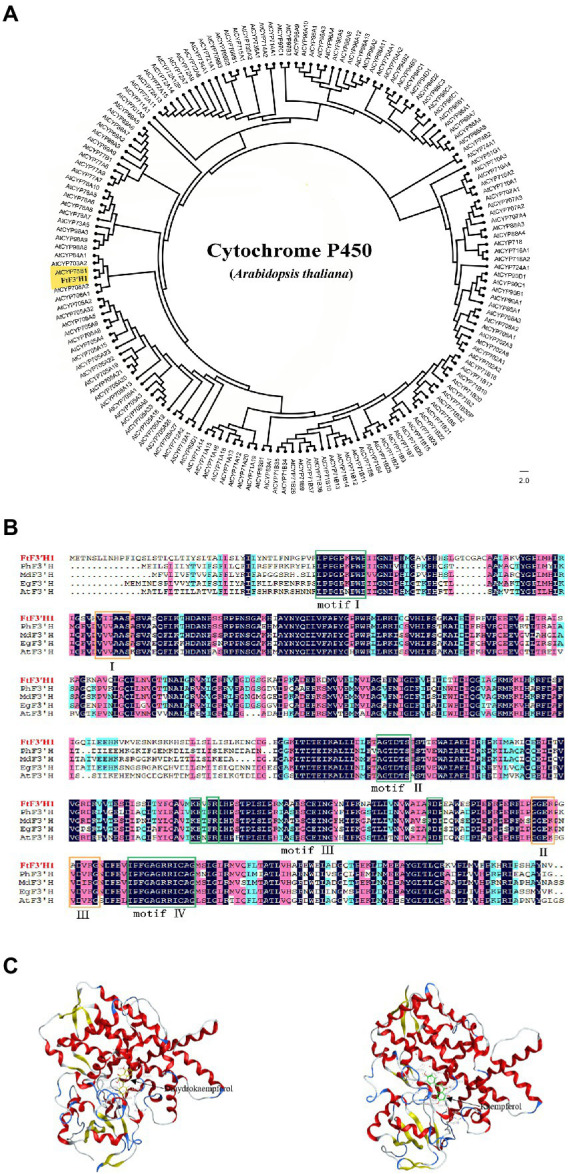
Structural features and phylogenetic analysis of the FtF3′H1 protein. **(A)** Molecular phylogenetic tree of F3′H proteins by the maximum likelihood method. **(B)** Sequence alignment of the deduced FtF3′H1 with F3′H proteins from other species, motifI (PPGPTPWP), motifII (FGAGRRICAG), motifIII (E-R-R), motifIV (AGTDTS), I (VVVAAS), II (GGEK), III (VDVKG). GenBank accession numbers for the proteins in the alignment are as follows: *Petunia* (AAD56282.1), *Malus* (ACR14867.1), *Eustoma grandiflorum* (BAP94456.1), *Arabidopsis thaliana* (CAB62611.1). **(C)** Docking prediction for FtF3′H1 protein with Dihydrokaempferol and Kaempferol.

Analysis of chromosome location showed that the *FtF3′H1* was located on chromosome 8 in TB (Supplementary Figure S2A). Sequence analysis revealed that *FtF3′H1* contained three exons and two introns, encoding a peptide containing 534 amino acids (Supplementary Figure S2C). The deduced protein molecular formula is C_2655_H_4212_N_724_O_755_S_23_, with a predicted relative molecular weight of 59.09 kDa and the theoretical *p*I of 7.69. Compared with the P450 proteins of *Arabidopsis*, results revealed that FtF3′H1 closely related to AtCYP75B1, which was the only gene encoding F3′H protein and regulated anthocyanin accumulation in *Arabidopsis* (Figure 2A). Basing on the characterized plant F3′Hs, the evolutionary tree showed that FtF3′H1 was classified into a member of the CYP75B subfamily (Supplementary Figure S2B). Furthermore, sequence alignment exhibited that FtF3′H1 had a high homology (about 80%) with F3′H sequence from *A. thaliana*, sweet potato, *Malus domestica*, and *Petunia hybrida* (Figure 2B). In addition, FtF3′H1 contains four typically conserved motifs of cytochrome P450 and three specifically conserved motifs of F3′H protein, suggesting that FtF3′H1 might have similar biological function in other species. Importantly, the interaction analysis emphasized that FtF3′H1 successfully docked to the substrates of dihydrokaempferol and kaempferol (Figure 2C). Altogether, the above data systematically revealed the molecular features of *FtF3′H1* and suggested that *FtF3′H1* might be involved in the regulation of flavonoid biosynthesis in TB.

### Expression pattern of *FtF3′H1* in TB

**Figure 3:**
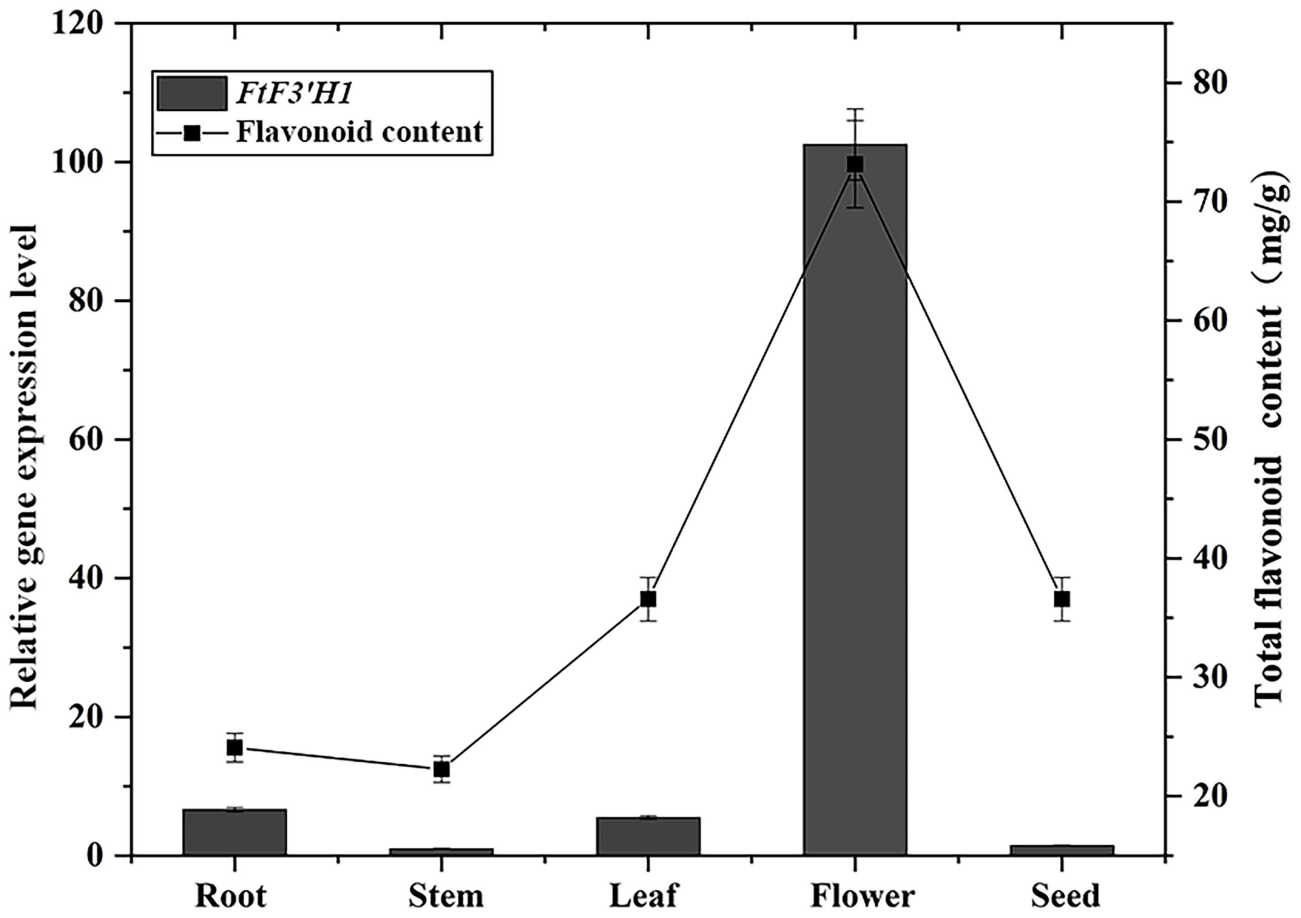
Relationship between flavonoid content and *FtF3′H1* transcript level in different tissues of TB at flowering stage. The expression of *FtF3′H1* was determined by qPCR. *FtH3* gene was used as the reference gene. Determination of total flavonoids of TB by UV spectrophotometer. The average value is calculated repeatedly by three times of technology. Error bars indicate standard deviations (± SD).

To further understand the relationship between *FtF3′H1* expression and flavonoid content in TB. The flavonoids were extracted and measured from roots, stems, leaves, flowers, and seeds of TB. Correspondingly, the expression pattern of *FtF3′H1* in five tissues was analyzed using qRT-PCR (Figure 3). The results showed a positive correlation between flavonoid content and *FtF3′H1* expression pattern (*R*^2^ > 0.95), which were highest in flowers, followed by roots, leaves, seeds, and stems in a decreasing order. Our results were also strongly correlated with the data obtained previously (Yao et al., 2017a; *R*^2^ > 0.95).

### Functional analysis of the *FtF3′H1* gene in the plants

To directly observe the effect of *FtF3′H1* on flavonoid biosynthesis, *FtF3′H1*-overproducing lines of *Arabidopsis*, and *tt7* mutant, *N. tabacum* and TB hair roots were generated (Supplementary Figure S4).

**Figure 4:**
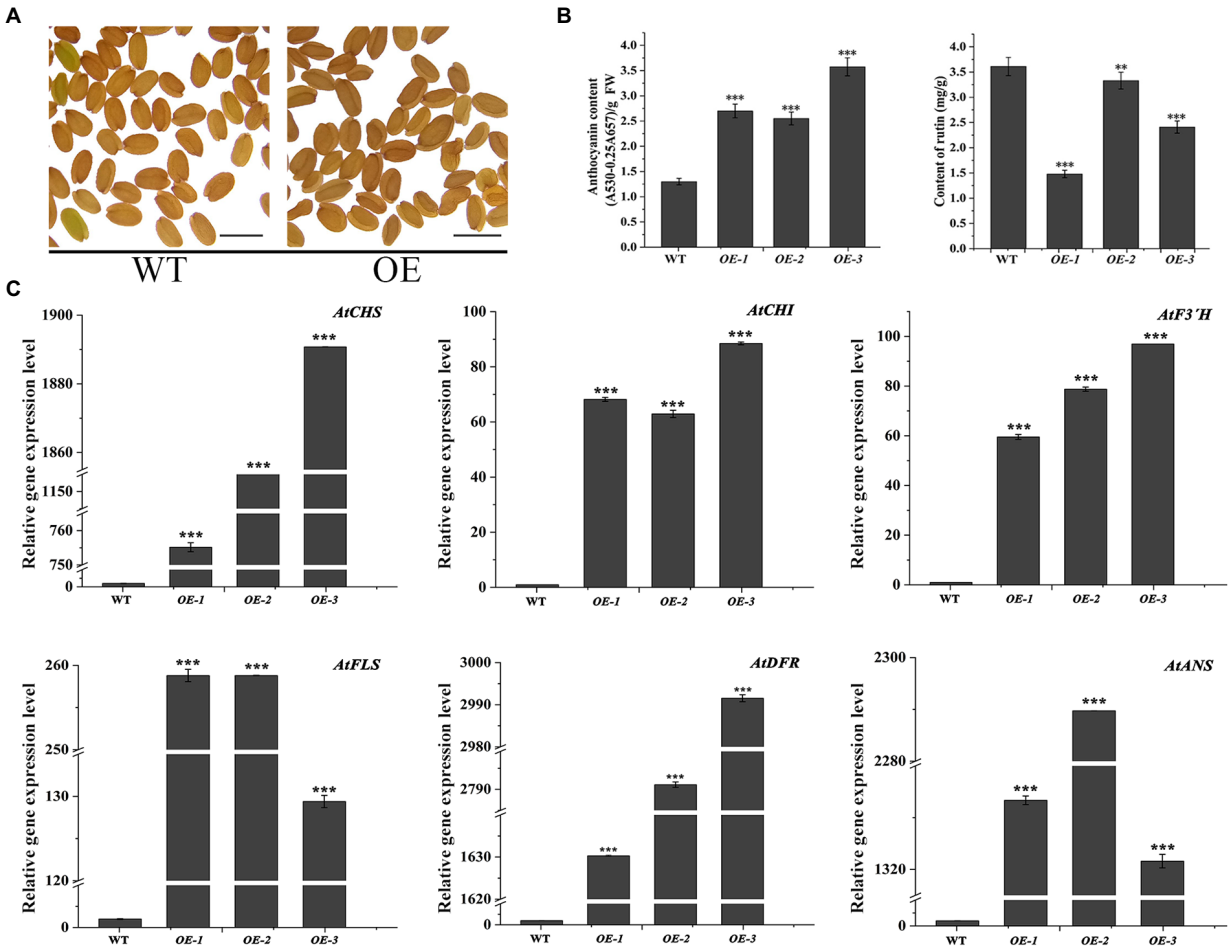
Overexpression of *FtF3′H1* gene in *Arabidopsis thaliana*. **(A)** Seed phenotypes of transgenic *A. thaliana* overexpressing *FtH3′H1* (OE) and WT (scale bar = 0.05 cm). **(B)** Determination of anthocyanin and rutin content in transgenic *Arabidopsis* lines OE-1, OE-2, and OE-3. WT is the control. Each value represents the mean of three replicates, and error bars indicate ± SD. ^**^p < 0.01 and ^***^p < 0.001. **(C)** qPCR analysis of the expression levels of anthocyanin biosynthesis genes in *A. thaliana* with the *FtF3′H1* overexpression. *Atactin2* was used as the reference gene. Results represent mean values ± SD from three biological replicates. ^*^p < 0.05, ^**^p < 0.01 and ^***^p < 0.001.

**Figure 5:**
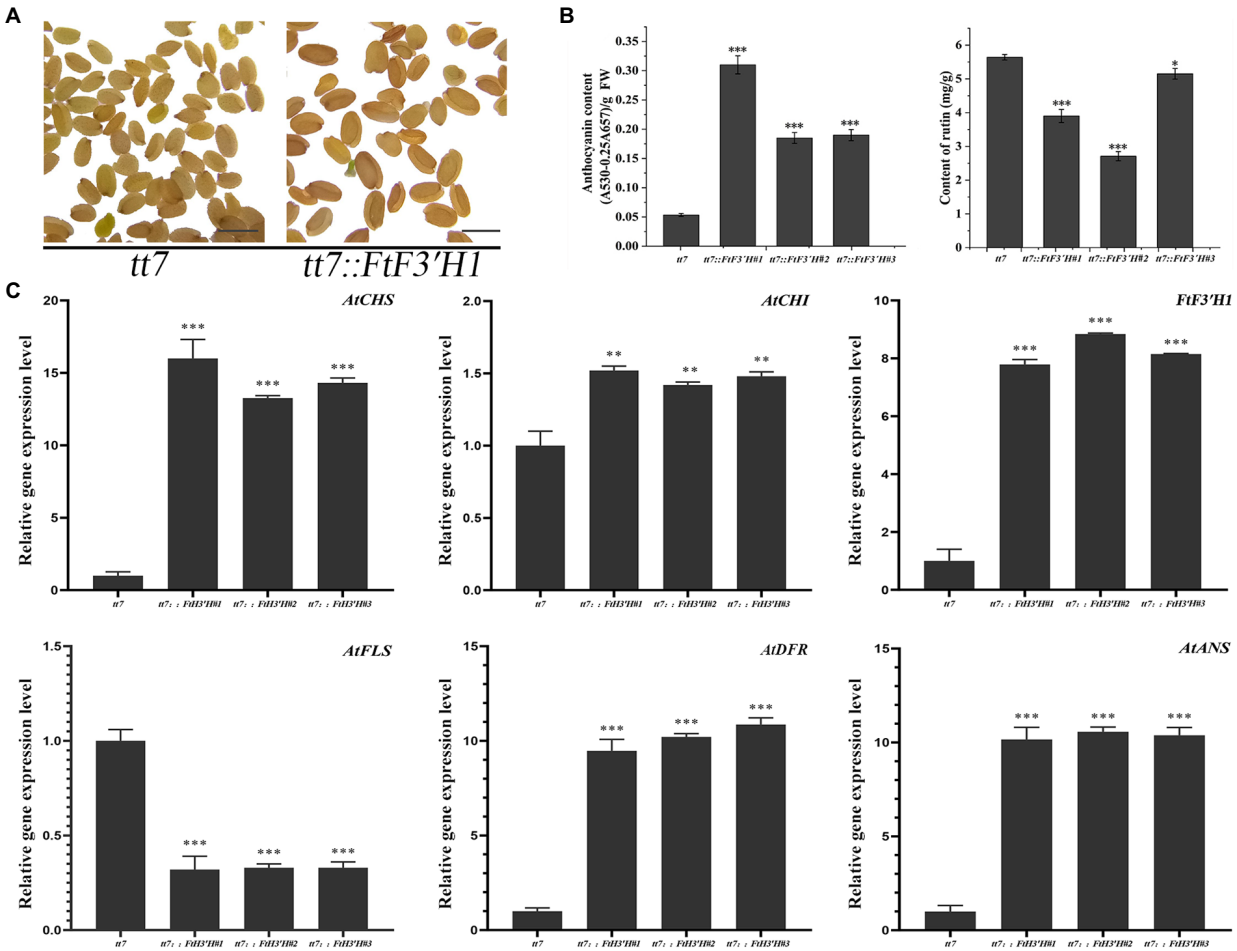
The original phenotype was restored after complementation of *FtH3′H1* in *tt7*. **(A)** The seed coat color of *A. thaliana tt7* with or without *FtH3′H1* (scale bar = 0.05 cm). **(B)** Determination of anthocyanin and rutin content in the corresponding *Arabidopsis* as indicated. Each value represents the mean of three replicates, and error bars indicate ± SD. ^*^p < 0.05, ^**^p < 0.01 and ^***^p < 0.001. **(C)** Transcription level of genes related to the anthocyanin biosynthesis in transgenic materials. *Atactin2* was used as the reference gene. Results represent mean values ± SD from three biological replicates. ^*^p < 0.05, ^**^p < 0.01 and ^***^p < 0.001.

In the transgenic *Arabidopsis* lines (OE1 ~ 3), the seeds visually showed deeper pigmentation than WT (Figure 4A), which might be attributed to the increased anthocyanin from its active biosynthesis. Data showed that the average anthocyanin content was 2.94 mg/g (fresh weight, FW) and significantly increased than WT (*p* < 0.05), while the rutin content was significantly reduced (*p* < 0.05; Figure 4B). Accordingly, the expression levels of flavonoid synthesis related gene, including *AtCHS*, *AtCHI*, *AtF3′H*, *AtFLS*, *AtDFR*, and *AtANS*, were upregulated (*p* < 0.05). Interestingly, the expression levels of *AtDFR* and *AtANS*, two key enzyme genes in the anthocyanin synthesis branch, were upregulated by thousand-folds, much higher than that of *AtFLS* in the flavonol synthesis pathway. The result might be the main reason for the increase in anthocyanin and decrease in rutin in OE1 ~ 3 (Figure 4C; *p* < 0.05). Similarly, in the *tt7* mutant *Arabidopsis* lines (*tt7::FtF3′H*1-3), FtF3′H1 overexpression restored the seeds color of *tt7* mutants (Figure 5A), increased anthocyanin and reduced rutin levels (Figure 5B), and upregulated expression of 5 genes involved in flavonoid synthesis (Figure 5C). Furthermore, the downregulation of *AtFLS*’s expression in *tt7* mutant demonstrated that *FtF3′H1* significantly enhanced the metabolic strength of anthocyanin synthesis branch in *Arabidopsis* (Figure 5C).

**Figure 6:**
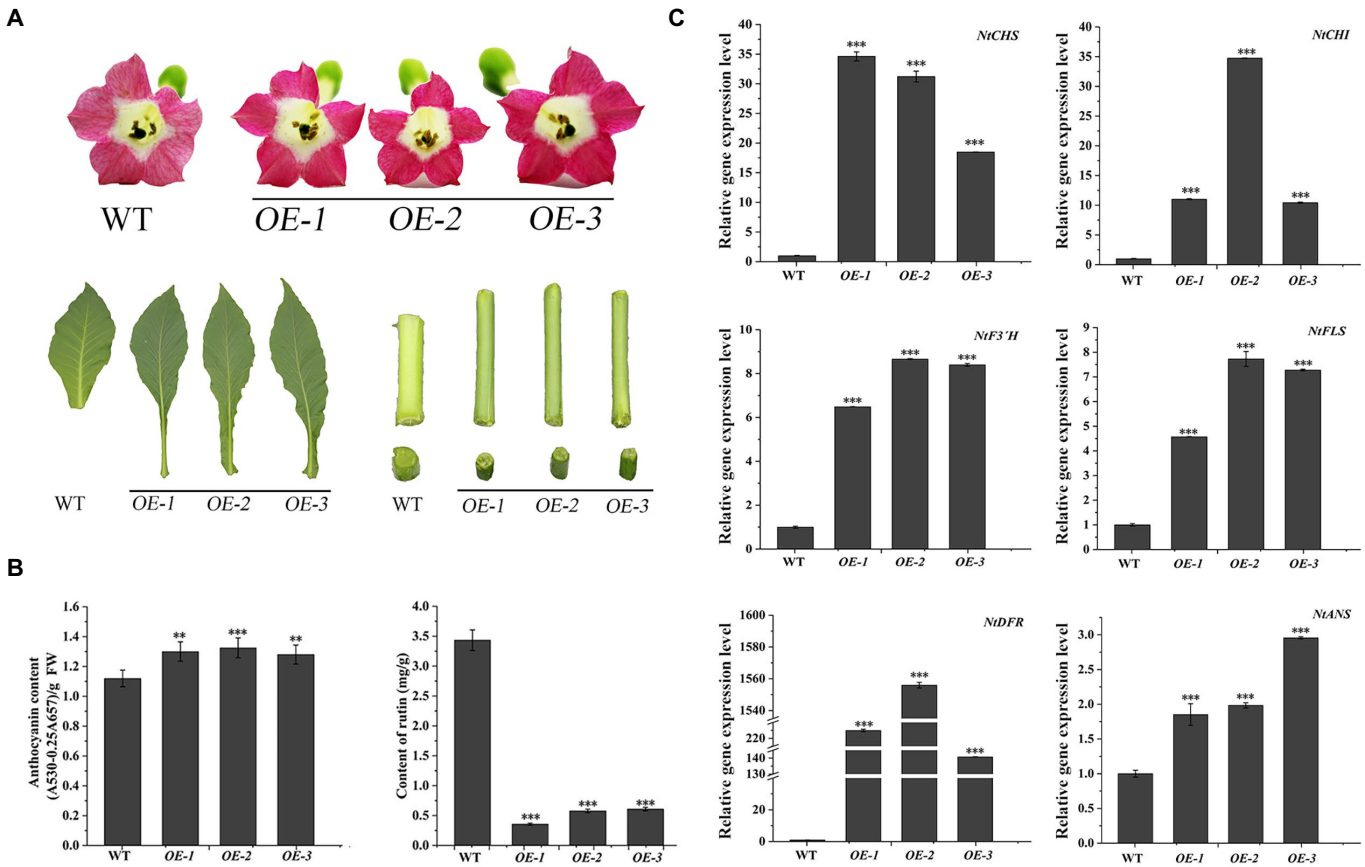
Overexpression of *FtF3′H1* gene promotes the accumulation of anthocyanins and inhibits the accumulation of rutin in *N. tabacum*. **(A)** Flowers petal pigmentation, leaf and stem growth in transgenic *N. tabacum* lines OE-1, OE-2 and OE-3. WT is the control. **(B)** Anthocyanins and rutin contents in transgenic *N. tabacum*. The average value is calculated repeatedly by three times of technology, and error bars indicate ± SD. ^**^p < 0.01 and ^***^p < 0.001. **(C)** Detection of transcriptional levels of flavonoid biosynthetic genes in transgenic *N. tabacum*. *Ntβ-actin* gene are used as a reference in *N. tabacum*. The average value is calculated repeatedly by three times of technology, and error bars indicate ± SD. ^*^p < 0.05, ^**^p < 0.01 and ^***^p < 0.001.

**Figure 7:**
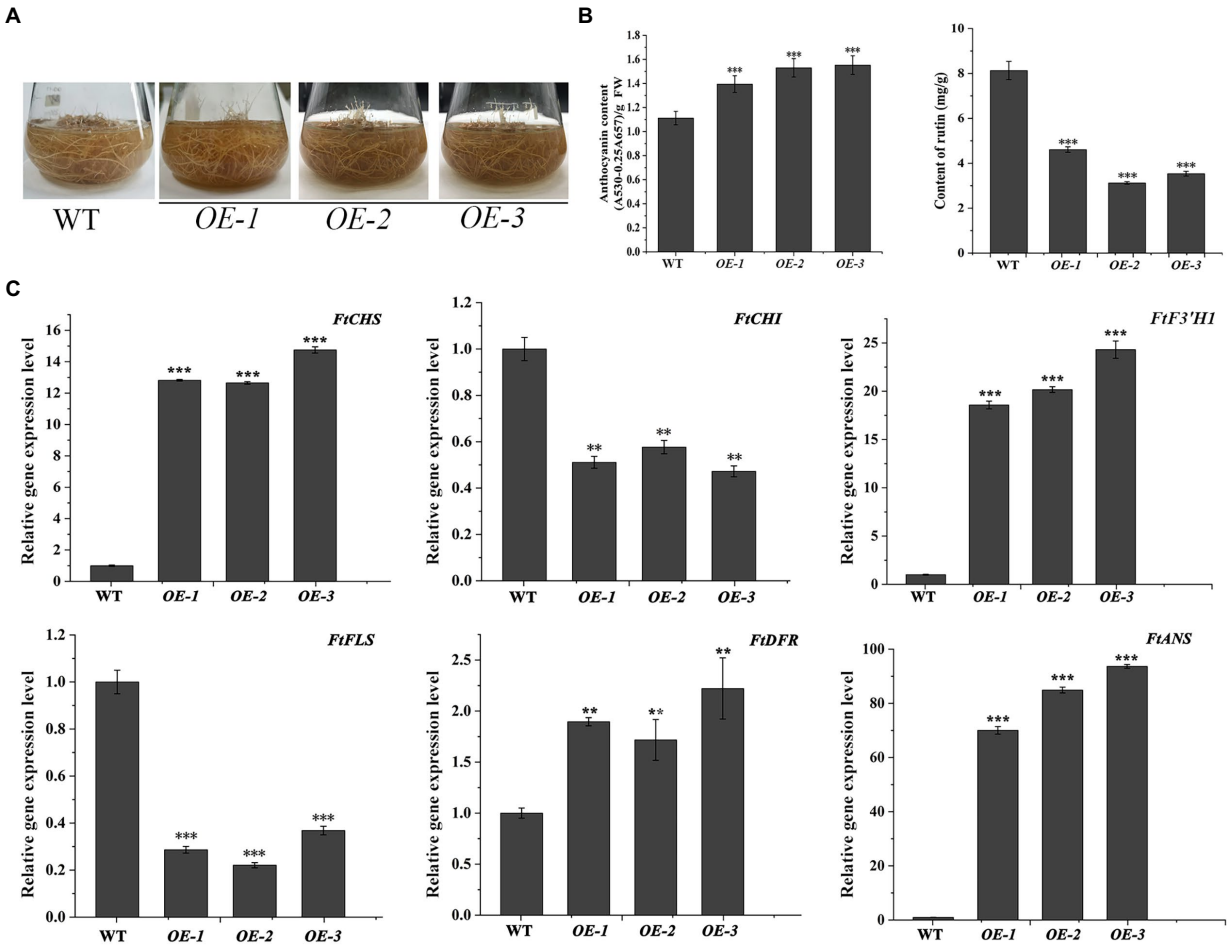
Overexpression of *FtF3′H1* in TB hairy roots. **(A)** Phenotypic comparison between WT and transgenetic TB hairy roots overproducing *FtF3′H1* (OE-1, OE-2 and OE-3). **(B)** Determination of anthocyanin and rutin contents in hairy roots. The average value is calculated repeatedly by three times of technology. Error bars represent ± SD. ^**^p < 0.01 and ^***^p < 0.001. **(C)** Expression analysis of genes associated with the anthocyanin biosynthesis in TB hairy roots by qPCR. *FtH3* gene is the reference gene. The average value is calculated repeatedly by three times of technology. Error bars indicate ± SD. ^*^p < 0.05, ^**^p < 0.01 and ^***^p < 0.001.

The transgenic *N. tabacum* lines (OE1 ~ 3) were used to confirm the biological feature of *FtF3′H1*. Compared to WT, *FtF3′H1* transgenic *N. tabacum* leaves were longer and narrower, and the flowers color were redder (Figure 6A). The core enzyme genes of flavonoid biosynthesis were significantly upregulated (*p* < 0.05), and the *NtDFR* gene was most strongly expressed (Figure 6C; *p* < 0.05). To accurately validate the effects of *FtF3′H1* in TB, TB hairy root lines overexpressing *FtF3′H1* (OE1 ~ 3) were generated (Figure 7A). Consistent with the results in transgenic *A. thaliana* (Figure 5B) and *N. tabacum* (Figure 6B), pigmentation in TB hairy roots derived from anthocyanin accumulation, accompanied by a decrease in rutin content (Figure 7B; *p* < 0.05). qRT-PCR assays showed an increased expression levels of *FtCHS*, *FtF3′H1*, *FtDFR*, and *FtANS*, whereas decreased levels of *FtCHI* and *FtFLS* (Figure 7C). Taken together, *FtF3′H1* is a key enzyme gene and branch switch for the metabolic pathway of flavonoids synthesis, and its overexpression can lead the metabolic flow to anthocyanin synthesis.

## Discussion

Tartary buckwheat is an annual herb of the dicotyledonaceae family and as a homology resource for medicine and food because of its enriched flavonoids. Flavonols and anthocyanins are the main flavonoids in TB, and they are produced from different metabolic branches in the same metabolism pathway. The distribution of anthocyanin and flavonol in TB has always been a complicated issue. Therefore, it is a key task to identify the switch gene functioning in flavonoid metabolism pathway from TB. In this study, *FtF3′H1* was shown to increase the content of anthocyanin and decrease the rutin levels in TB. It strongly indicates that FtF3′H1 might be a perspective gene for the quality improvement of TB by increasing anthocyanins *via* genetic engineering.

In the CYP75 subgroup of plant P450 family, molecular characterization is an effective way to identify the *F3′H* gene. In this study, FtF3′H1 protein are highly homologous to the F3′H proteins in *Vitis vinifera* (Jeong et al., 2006; Jochen Bogs et al., 2006), *Malus* (Hutabarat and Halbwirth, 2020), and *Petunia* (Brugliera et al., 1999). Unsurprisingly, the FtF3′H1 contained four cytochrome P450 conserved motifs (PPGPTPWP, FGAGRRICAG, AGTDTS, and E-R-R) and three characteristic motifs of F3′H (VVVAAS, GGER/K, and VDVKG; Figure 2B). Among them, PPGPTPWP is a proline-rich region, which is the main conserved domain and responsible for signal anchoring (Murakami et al., 1994). FGAGRRICAG, a heme-binding region containing conserved cysteine residues, can bind iron ions as well as carbon monoxide (Chika et al., 2001). AGTDTS is a molecular oxygen-binding domain that is essential for catalytic reactions (Baba and Ashraf, 2019). E-R-R, a domain that stabilizes protein structures (Zabala and Vodkin, 2003; Seitz et al., 2006). Each of the three characteristic motifs can be the F3′H marker, such as GGER/K, which can be used as an important marker to distinguish F3′H from F3′5′H and other P450s (Zabala and Vodkin, 2003; Seitz et al., 2006).

In order to explore how *FtF3′H1* affects the biosynthesis of flavonoids, a soluble recombinant protein FtF3′H1 tagged with GST was induced in *E. coli* and purified. Subsequently, the enzyme activity was determined by feeding substrates (kaempferol and dihydrokaempferol) and analyzed by HPLC. Unfortunately, the theoretical products including quercetin and dihydroquercetin were not able to be detected in the reaction (Supplementary Figure S3). This failure may be due to the lack of cytochrome P450 reductase in the prokaryotic expression system, which is essential for the delivery of electrons (Sevrioukova et al., 1999). Additionally, the deficiency of endoplasmic reticulum membrane structures in the system also prevents the FtF3′H1 from fulfilling its activity (Sevrioukova et al., 1999). Differently, in *Catharanthus roseus* (Hotze et al., 1995) and *Camellia sinensis* (Guo et al., 2019), the F3′H enzyme activities were measured by excising the membrane-bound region of cytochrome P450 enzyme and cytochrome P450 reductase. In the future, we will use different strategies to further analyze the catalytic activity of FtF3′H1 *in vitro*.

Previous studies have shown that the transcription levels of *F3′Hs* are significantly related to the synthesis of flavonoids and accumulation of pigments in plants. During fruit development and coloring of *Vitis vinifera*, it was found that the activation of the flavonoid pathway resulting in the total flavonoid and anthocyanin accumulation had a strong correlation with the *F3′H* expression (Jeong et al., 2006). Similar conclusions were drawn in *Arabidopsis*, *Camellia sinensis*, *Sorghum bicolor*, and other plants (Forkmann and Martens, 2001; He et al., 2020a; Cai et al., 2022; Hildreth et al., 2022). Interestingly, in Korean varieties of white, black, and red rice (*Oryza sativa*), 2 *F3′Hs* (*CYP75B3* and *CYP75B4*) showed different expression patterns. *CYP75B3* and *CYP75B4* were mainly expressed in the developing seeds of black rice, but not in those of white and red rice. The expression levels of *CYP75B4* were much higher than those of *CYP75B3* in the developing seeds, leaves, and roots of white rice. The results suggested that different copies of the *F3′Hs* might regulate the synthesis and accumulation of different types of flavonoids in plants through changes in expression pattern. Consistently, the same phenomenon was also observed in this study. Based on the transcriptome and qRT-PCR data of flowering TB, the accumulation of total flavonoids may be mainly due to the high expression of *FtF3′H1* rather than its homologous copy *FtF3′H2* (Supplementary Figure S1), and it shows a significantly positive correlation with the *FtF3′H1* expression (*R*^2^ = 0.95; Figure 3).

In the metabolic pathway of flavonoids, the function of F3′H enzyme is to catalyze the hydroxylation of the 3rd C atom in different flavonoid subtracts on the B ring, such as dihydrokaempferol, kaempferol, pelargonidin and so on. In this biochemical process, FLS, DFR, ANS, and F3′H may share the same subtracts and compete with each other, leading to metabolic flow favoring anthocyanin or flavonol branch (Martínez-Lüscher et al., 2014; Sakuta et al., 2021; Shi et al., 2021). In some cases, increased expression of *F3′H*s have been shown to improve anthocyanin accumulation in some plants *via* shifting flavonoid metabolism toward the anthocyanin branch. In apple (*Malus × domestica*), expression of *MdFLS* is down-regulated during late stages of fruit development, which can be attributed to high levels of expression of *MdF3′H* genes and the competition of MdF3′H and MdFLS for the same substrate (Han et al., 2010). Wu reported that overexpression of the *GbF3′H* (*Ginkgo biloba*) could enhance the anthocyanin synthesis and accumulate more red-colored pigments in leaves of transgenic *Populus* (Wu et al., 2020). In another cases, silencing or mutation of the *F3′H* gene may lead to the enhancement of flavonol synthesis branch and the reduction of anthocyanin content. In *Paeonia suffruticosa*, an indel in *F3′H* caused the upregulation of *FLS* and drastically reduced the anthocyanin content in acyanic petals (Zhang et al., 2020). Our data from transgenic lines, including *Arabidopsis*, *Arabidopsis tt7* mutants, *N. tabacum*, and TB hairy roots, showed that the overexpression of *FtF3′H1* caused an increase in anthocyanin synthesis and decreased the content of rutin. These results are consistent with the reports in Chinese Cabbage (*Brassica rapa* L. subsp. pekinensis; He et al., 2020a,b, Park et al., 2021), *O. sativa* (Shih et al., 2008), and *Paeonia suffruticosa* (Shih et al., 2006; Zhang et al., 2020; Nitarska et al., 2021). In our experiments, *FtF3′H1* increased the accumulation of anthocyanin, which may due to the higher expression abundance of *FtF3′H1* than *FtFLS*s or *FtF3′H1* has a stronger substrate preference for anthocyanin branch.

In conclusion, we successfully cloned *FtF3′H1* with a complete domain from TB, characterized its molecular features and biological function *in vivo*. Notably, this study validated that *FtF3′H1* is not only a key enzyme gene, but more essentially acting as a metabolic branch switch for TB flavonoid synthesis.

## Data availability statement

The original contributions presented in the study are included in the article/Supplementary material, further inquiries can be directed to the corresponding author.

## Author contributions

CL and KY wrote the first draft of the paper. JY performed most of the experiments. HW helped the writing language of the draft. HC provided modification information in the revised version of manuscript. HZ and QW participated in the preparation of the manuscript. All authors contributed to the article and approved the submitted version.

## Funding

This work was supported by the National Key R&D Program of China (2021YFD1200105) and National Natural Science Foundation of China (31871698).

## Conflict of interest

The authors declare that the research was conducted in the absence of any commercial or financial relationships that could be construed as a potential conflict of interest.

## Publisher’s note

All claims expressed in this article are solely those of the authors and do not necessarily represent those of their affiliated organizations, or those of the publisher, the editors and the reviewers. Any product that may be evaluated in this article, or claim that may be made by its manufacturer, is not guaranteed or endorsed by the publisher.

## Supplementary material

The Supplementary material for this article can be found online at: https://www.frontiersin.org/articles/10.3389/fpls.2022.959698/full#supplementary-material

## Footnotes

^1^ http://gsds.cbi.pku.edu.cn/

^2^ https://web.expasy.org/protparam/
