# Corrigendum: Tartary buckwheat *FtF3′H1* as a metabolic branch switch to increase anthocyanin content in transgenic plant

**DOI:** 10.3389/fpls.2022.1056857

**Published:** 2022-11-17

**Authors:** Chenglei Li, Jingjing Yang, Kai Yang, Huala Wu, Hui Chen, Qi Wu, Haixia Zhao

**Affiliations:** College of Life Science, Sichuan Agricultural University, Ya’an, Sichuan, China

**Keywords:** Tartary buckwheat (*Fagopyrum tataricum*), metabolism, flavonoid biosynthesis, flavonoid 3′-hydroxylase, transgenic plants

In the published article, there was an error in the author list. Author Jingjing Yang was erroneously listed as the third author, but should be the second author and marked as sharing equal contribution and first authorship. The corrected author list appears below:

“Chenglei Li†, Jingjing Yang†, Kai Yang†, Huala Wu, Hui Chen, Qi Wu and Haixia Zhao*”

The authors apologize for this error and state that this does not change the scientific conclusions of the article in any way. The original article has been updated.

## Publisher’s note

All claims expressed in this article are solely those of the authors and do not necessarily represent those of their affiliated organizations, or those of the publisher, the editors and the reviewers. Any product that may be evaluated in this article, or claim that may be made by its manufacturer, is not guaranteed or endorsed by the publisher.

