# Supplementary material for: Tartary buckwheat FtF3′H1 as a metabolic branch switch to increase anthocyanin content in transgenic plant
Source: Front Plant Sci. 2022 Aug 25;13:959698. doi: 10.3389/fpls.2022.959698 (PMC9452690; doi:10.3389/fpls.2022.959698)
Supplement: Supplementary file 2 [file Data_Sheet_1.docx]

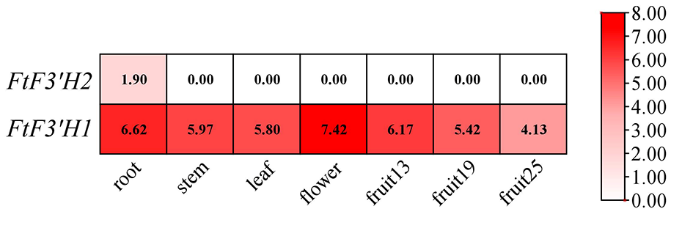


**Figure.S1**. Expression analysis of two *FtF3′H* genes in different tissues of Tartary Buckwheat. Fruit_-_13, fruit_-_19, fruit_-_25 represents seeds on the 13th, 19th, and 25th days after flowering, respectively.


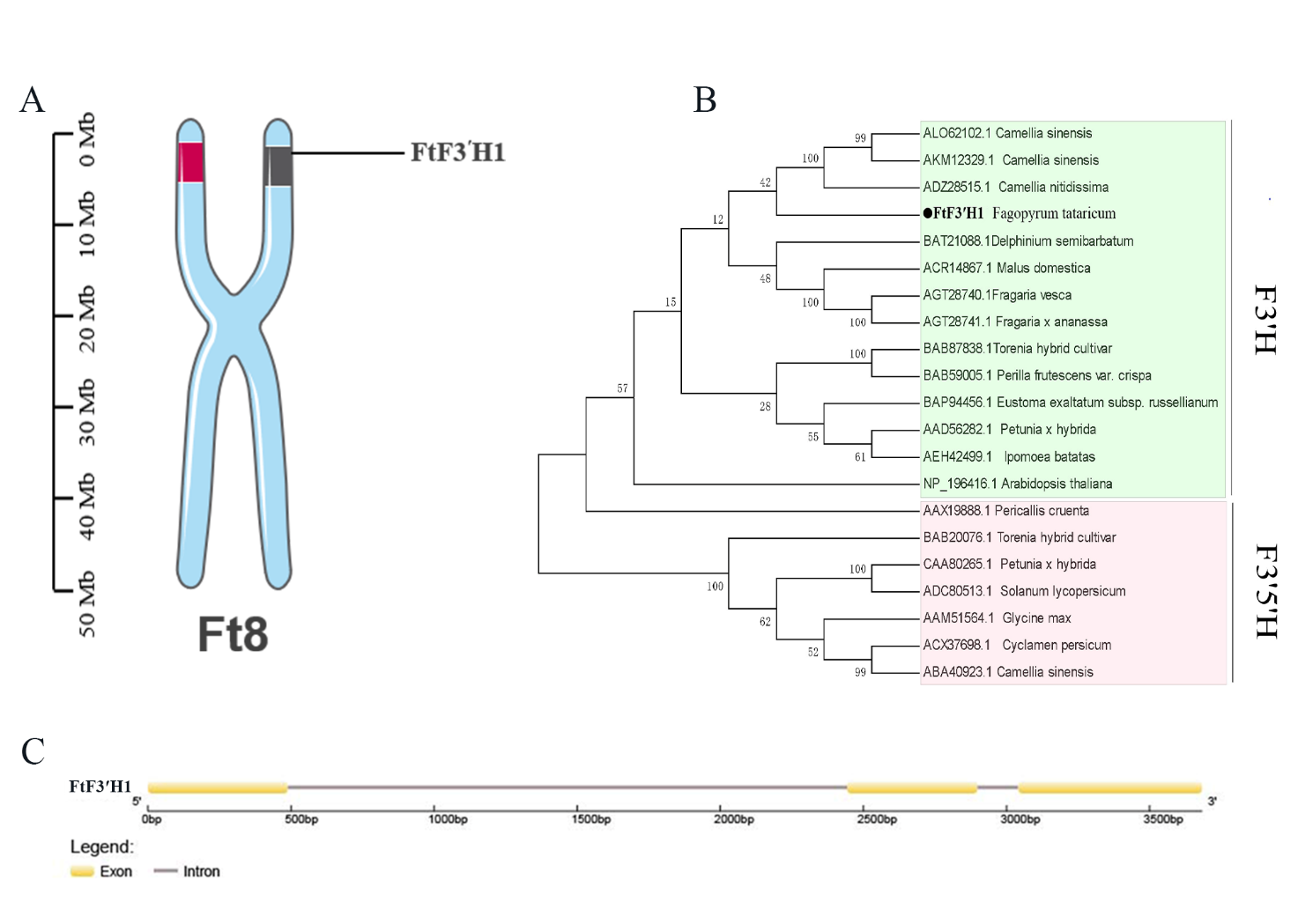


**Figure.S2**. Structures and phylogenetic analysis of the *FtF3′H1* gene. **(A)** Position of *FtF3′H1* gene on the TB chromosomes. **(B)** Evolutionary analyses of F3′H proteins were conducted in MEGA 7.0. **(C)** Structures of the *FtF3′H1* gene. Exons and introns are indicated by yellow boxes and grey lines, respectively.


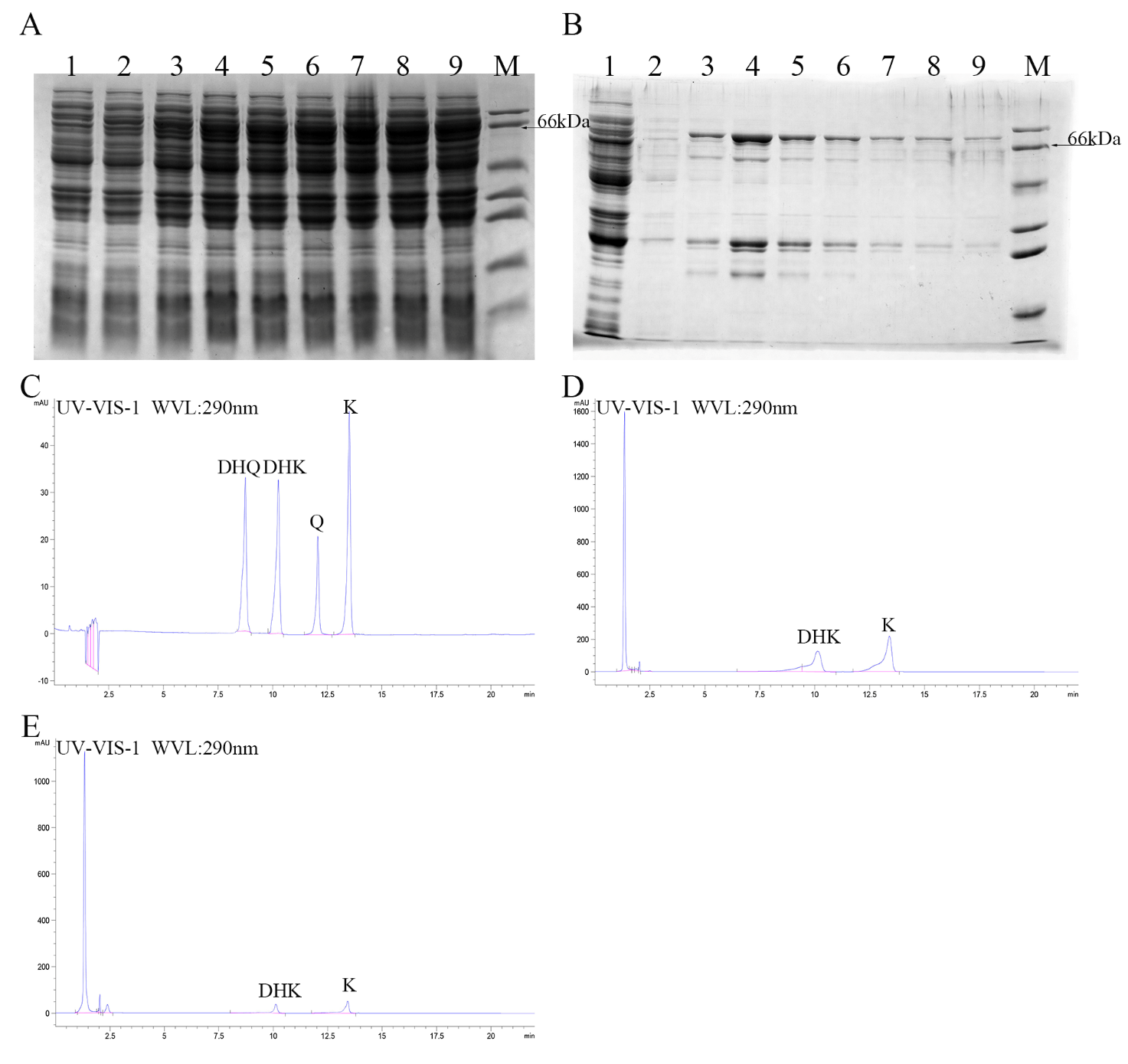


**Figure.S3**. In vitro detection of pGEX-4T-1-FtF3′H protein activity by substrate feeding. **(A)** The results of pGEX-4T-1-*FtF3′H* by inducing expression in *E. coli* BL21, M: protein standard molecular weight; 1-9: pGEX-4T-1-FtF3′H recombinant proteins induced by IPTG for 0、1、2、3、4、5、6、7、8 h. **(B)** The results of isolation and purification of pGEX-4T-1-*FtF3′H* by inducing expression in *E. coli* BL21, M: protein standard molecular weight; 1: cell lysate; 2: precipitation; 3: supernatant; 4-9: eluate 4-9. **(C)** HPLC standard ( Q=quercetin, DHQ=dihydroquercetin, K=kaempferol, DHK=dihydrokaempferol) results. **(D)** Recombinant protein pGEX-4T-1-*FtF3′H* after sonication, the substrates are kaempferol, dihydrokaempferol. **(E)** Unsonicated recombinant protein pGEX-4T-1-*FtF3′H*, the substrates are kaempferol, dihydrokaempferol.


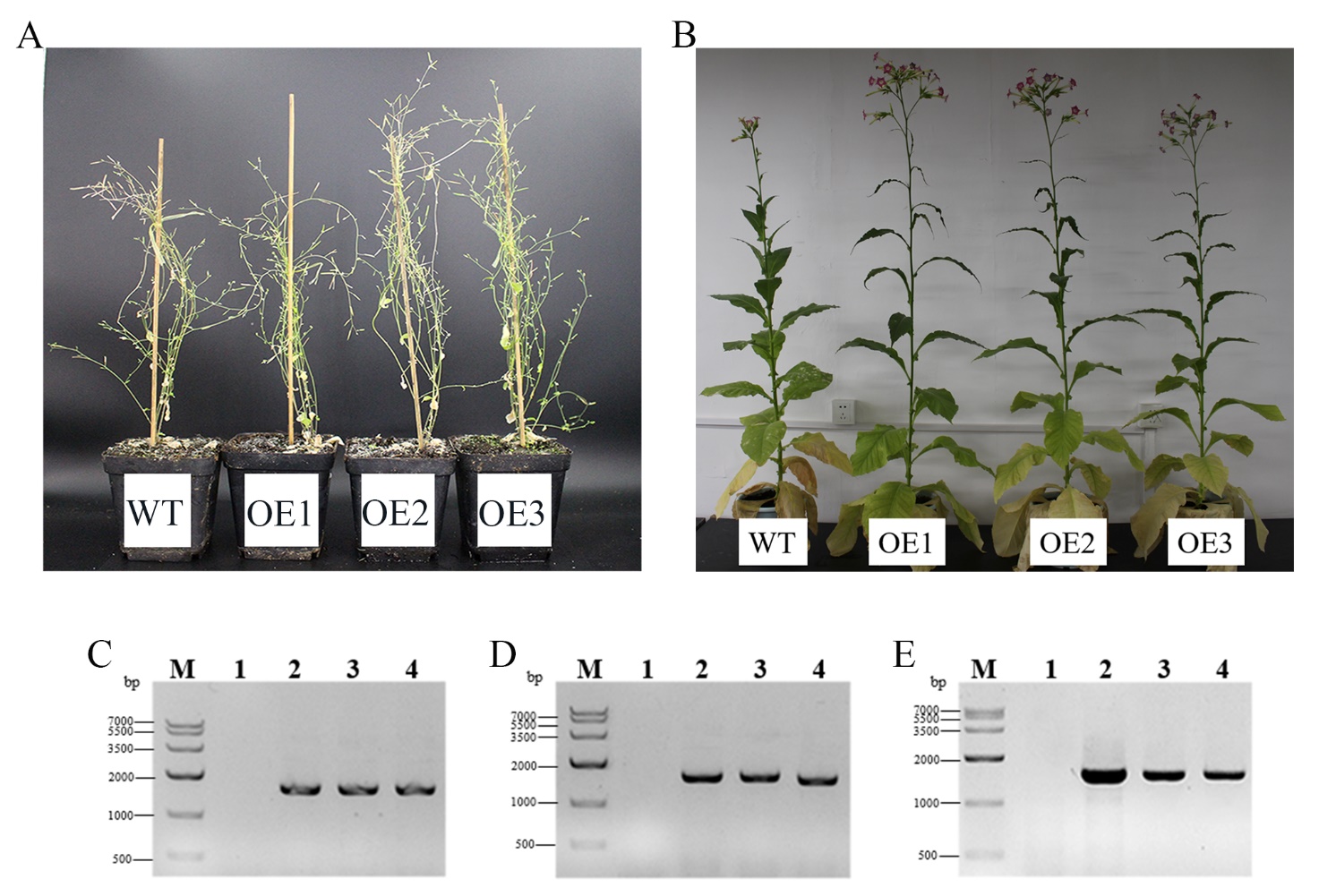


**Figure.S4**. Positive identification of transgenic plants. (**A)**, (**B)**: Observation of phenotype of transgenic *Arabidopsis* and *N. tabacum*. (**C)**, (**D)**, (**E)**: Represents the PCR positive identification results of transgenic *Arabidopsis*, *N. tabacum*, and TB hairy root plants, respectively. M: DNA standard molecular weight, 1: WT, 2-4: OE1-3.
